# Microbiome Diversity, Persistence, and Potential Symbiont Dynamics in *Placobdella* Leeches from American Alligators

**DOI:** 10.1007/s00248-026-02897-x

**Published:** 2026-10-08

**Authors:** Logan M. Morris, Miriam Boucher, Charlie Service, James T. Anderson, Thomas R. Rainwater, Benjamin B. Parrott, Shana K. Goffredi

**Affiliations:** 1 Biology Department at Occidental College, Los Angeles, CA USA; 2 Belle W. Baruch Institute of Coastal Ecology and Forest Science, Clemson University, Georgetown, SC USA; 3 Tom Yawkey Wildlife Center, Georgetown, SC USA; 4 Savannah River Ecology Laboratory, University of Georgia, Aiken, SC USA; 5 Eugene P. Odum School of Ecology, University of Georgia, Athens, GA USA

**Keywords:** *Placobdella*, Alligator, Leech, Blood-feeding, Microbiome, *Reichenowia*

## Abstract

*Placobdella* leeches are common ectoparasites of reptiles that rely on microbial symbionts to supplement the nutritional limitations of blood feeding. Although the primary symbiont *Reichenowia* is documented, little is known about the broader microbial community or ecological factors that influence *Placobdella* microbiome assembly. Here, we characterized the whole-body microbiomes of seven *Placobdella* clades from American alligators (*Alligator mississippiensis*) across the southeastern United States. Sampling revealed four *Placobdella* lineages not previously reported from alligators, including several undescribed species. *Placobdella* microbiomes, based on 16S rRNA gene sequencing, were dominated by Actinobacteriota, Alphaproteobacteria, Bacteroidota, and Pseudomonadota (the former Betaproteobacteria). Although microbiome composition was not strongly structured by leech clade, geography, or habitat type, location-specific bacterial signatures were observed. Captivity experiments resulted in significant declines in microbial diversity, and persistence of four bacterial taxa. Comparisons between leech microbiomes and microbial communities on alligator skin revealed limited overlap. Additionally, while a distinct *Reichenowia* lineage was generally associated with each *Placobdella* species, multiple low-abundance *Reichenowia* variants were detected within individuals, and an identical *Reichenowia* variant occurred in sympatric *Placobdella* species. Together, these results identify a conserved candidate core microbiome in *Placobdella* and demonstrate that microbiome assembly in blood-feeding leeches reflects persistent symbiotic interactions and ongoing environmental acquisition.

## Introduction

Parasites, including hematophagous leeches, play important ecological roles by modifying the physiology and behavior of their hosts, both directly and indirectly [37, 51]. For example, leeches within the genus *Placobdella* have been implicated as likely vectors of the apicomplexan blood parasite *Haemogregarina crocodilinorum* in the American alligator *Alligator mississippiensis* [22], which can result in anemia when infections become severe [2, 21]. Little is known about how leeches directly affect alligator fitness, although heavy infestations can cause lesions and juvenile crocodilians may be particularly susceptible [48]. American alligators are widely distributed throughout freshwater and brackish habitats of the southeastern United States and host a diverse assemblage of parasites, including *Placobdella* leeches [12, 18, 48]. Leech load can vary seasonally for alligators, but some individuals carry a significant parasite burden, for example, hosting hundreds of leeches in their mouths. While their thick skin is thought to offer some protection, leeches are commonly observed attached to the external surfaces between scales, in their armpits, and on their toes [48]. Due to their mobility among aquatic habitats [34, 44], alligators may facilitate exposure of *Placobdella* leeches to diverse environmental microbial communities.

Leeches within the genus *Placobdella* are specialized reptile ectoparasites within the family Glossiphoniidae. The genus includes 25 species that inhabit a variety of freshwater environments, including rivers, lakes, ponds, wetlands, and swamps [8, 9]. While some species are opportunistic generalists, many associate with specific hosts. Three described species *(P. siddalli*,* P. multilineata*, and *P. papillifera*), and one currently undescribed *Placobdella* species (*Placobdella* sp. 3) have been reported on American alligators [8, 9, 14, 19]. Recent molecular phylogenetic studies suggest that *Placobdella* diversity is underestimated, with evidence for cryptic and undescribed lineages across North American freshwater systems [9, 13, 28, 36].

Like all blood feeders, *Placobdella* must contend with B vitamin deficiencies, red blood cells that are difficult to lyse and digest, and heme toxicity. The latter occurs during blood feeding because liberated heme is highly reactive and can cause oxidative cellular damage [29, 47]. While vertebrate blood provides a large, concentrated nutritional resource, B vitamins, which are crucial for many cell processes and metabolic functions, are scarce [11]. Blood feeders, such as *Placobdella* leeches, compensate for this lack of B vitamins by hosting bacterial symbionts [11]. In *Placobdella*, alphaproteobacterial symbionts in the genus *Reichenowia* are localized in sac-like organs known as mycetomes, attached to the esophagus, just behind the proboscis [43]. *Reichenowia* are thought to aid in the production of B vitamins and provide essential nutrients otherwise lacking in their diets [11, 23]. Previous research suggests that each *Placobdella* species harbors a lineage of endosymbiont that is passed down through vertical transmission from parent to offspring, although evidence for strict co-speciation has not been observed [11, 30, 43].

Although *Reichenowia* is a well-established symbiont of *Placobdella*, the broader microbial community associated with these leeches has received little investigation. This study is a molecular analysis of seven *Placobdella* clades from American alligators, some undescribed, from a wide geographic range. The factors shaping *Placobdella* microbiome composition were examined, including host lineage, geographic location, hydrologic habitat type, and potential exposure to alligator skin-associated microbial communities. The relative contributions of host identity and habitat to microbiome assembly were evaluated. In addition, we sought to characterize candidate core taxa shared across *Placobdella* lineages (operationally defined as the presence of an amplicon sequence variant (ASV) in at least 90% of the wild specimens, and those persisting in the population of captive specimens at > 5% relative abundance). If present, a stable core microbiome in *Placobdella* may provide a tractable model for understanding how host-associated microbiomes are assembled and maintained over time.

## Methods

### Sample Collection and Habitat Characterization

**Fig. 1:**
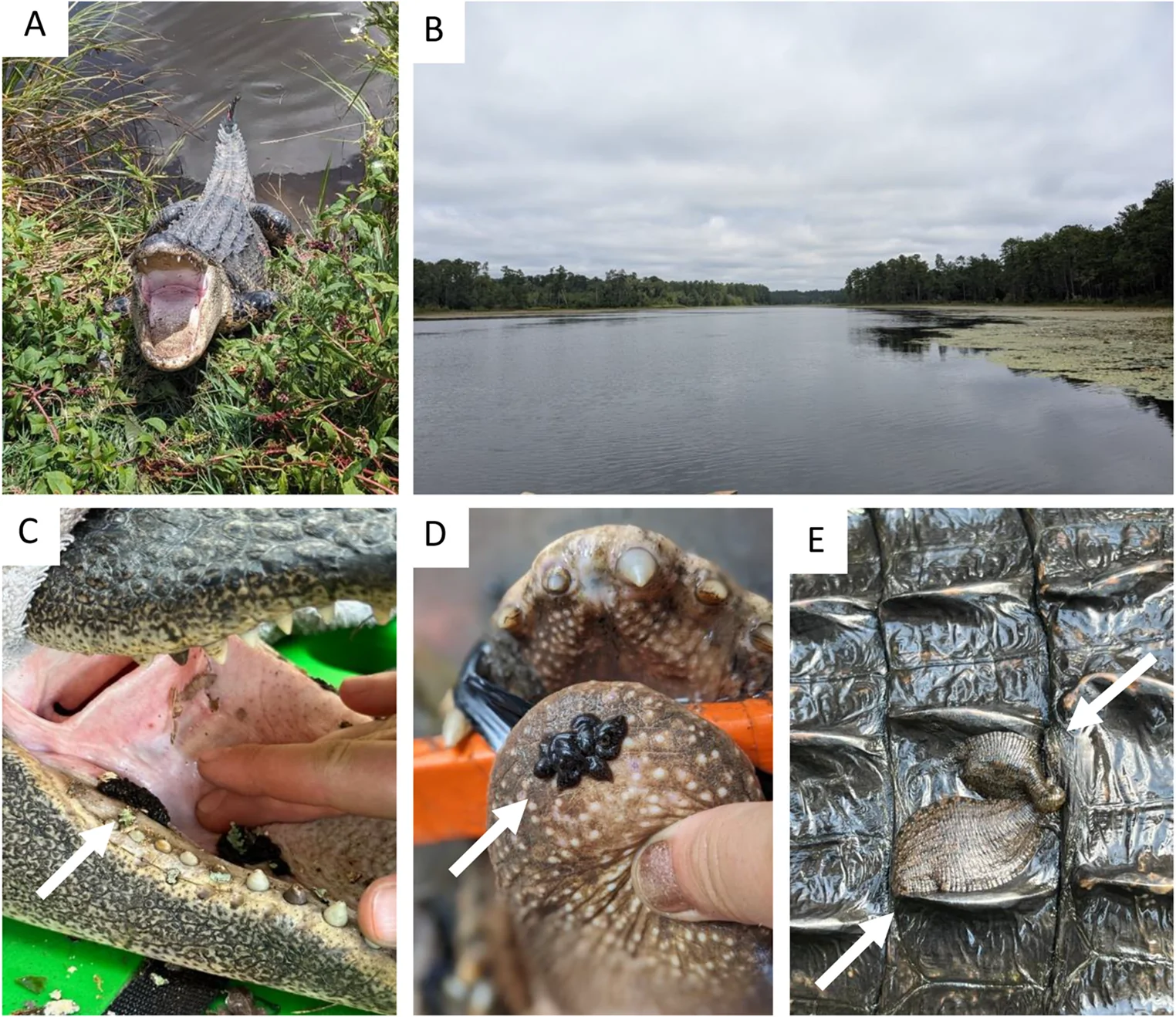
Specimen collection for this study. **A** An American alligator host (*Alligator mississippiensis*). **B** An example collection site, Par Pond, South Carolina. Leeches (arrows) were collected from the oral cavity (**C**), the top of the tongue (**D**), on the dorsal surface (**E**), and beneath the chin and forelimbs (not pictured). Leech body lengths ranged from approximately 5 mm to 5 cm

*Placobdella* leeches were collected in 2023–2025 from alligators in 12 locations throughout North Carolina, South Carolina, Georgia, Mississippi, Alabama, Texas, and Florida (Fig. 1; Table 1). Sampling effort varied among locations depending on alligator availability (*n* = 27) and leech abundance (*n* = 42). Leeches were sampled from both live-captured alligators, as well as from alligators legally harvested through state-managed public and private harvest seasons (MS, AL, TX, FL). Leeches were collected, using forceps and gentle force at the site of attachment, from the oral cavity, beneath the chin, on the dorsal surface, and beneath the forelimbs and placed in 95% ethanol [30]. For the 2025 collection, the attachment area on the alligator was also swabbed and swabs preserved in RNAlater (Thermo Fisher Scientific) for subsequent DNA extraction.

**Table 1:** Sampling locations across the southeastern United States including state, geographic coordinates, hydrologic classification, estimated surface area, collection dates, number of alligators sampled, and number of individual *Placobdella* specimens recovered from each site, listed by state from northeast to southwest

Site	State	Latitude	Longitude	Habitat	Surface Area (km2)ª	Collection Dates	# alligators sampled	# leeches recovered
Lake Ellis-Simon	NC	34.84187	-76.98825	Lake	4.45	16 Aug 2023, 20 Jul 2024	6	6
Lake Waccamaw	NC	34.30068	-78.55293	Lake	34.8	3 May 2023, 31 Jul 2023	2	2
Yawkey	SC	33.23946	-79.23247	Pool	0.59	5 Sep 2024	1	1
Par Pond	SC	33.30146	-81.51705	Lake	9.23	13 Jul 2023, 6 Aug 2025	2	8
Savannah NWR	SC	32.17921	-81.10580	River	n/a	3 Jul 2024, 7 Aug 2025	7	12
Savannah River	GA	32.17457	-81.15518	River	n/a	30 Jul 2024	3	4
Jekyll Island	GA	31.07152	-81.41529	Pool	0.02	14 Jun 2024	1	2
Lake George	FL	29.33641	-81.65669	Lake	176	14 Sep 2024	1	2
Mobile-Tensaw Delta	AL	30.67960	-87.93472	Pool	0.77	15 Aug 2024	1	1
Pearl River^b^	MS	32.58363	-89.84902	River	n/a	27 Aug 2023	1	2
Dam B	TX	30.86727	-94.18312	River	n/a	13 Sep 2023	1	1
Ox Bow	TX	28.11798	-97.39805	River	n/a	14 Oct 2023	1	1

^a^ estimated from Google Earth; ^b^ at Ross Barnett Reservoir

Alligators were captured using conventional techniques including neck nooses, snatch hooks [49, 50], and baited trip snares [32]. Captures occurred after sunset to coincide with peak nocturnal activity of alligators. Alligator sample collection was approved by Clemson University Animal Use Protocol #2022 − 0454 and permitted by the South Carolina Department of Natural Resources (permit # Rainwater 2024), Georgia Department of Natural Resources (permit # 1001742080), Savannah National Wildlife Refuge (permit # 2025-16), Florida Fish and Wildlife Conservation Commission (permit # SPGS-25-44), and Alabama Department of Conservation and Natural Resources (permit # 2024149244868680). Alligator sampling in North Carolina was conducted with biologists from the North Carolina Wildlife Resources Commission, requiring no permits. Alligator habitat categories were included to assess whether hydrologic connectivity and water flow influence *Placobdella* microbiome composition. If the sampling habitat was characterized by directional flowing water, it was considered a river. If the habitat was enclosed or isolated and consisted of mainly still water, it was identified as either a lake or a pool. Lakes and pools were distinguished using a 1 km² surface area threshold measured in Google Earth Pro, with lakes defined as > 1 km² and pools as ≤1 km² (Table 1). In total, there were five river, four lake, and three pool sampling locations.

A subset of leech specimens collected in 2025 was maintained alive to assess temporal changes in microbiome composition during captivity. After removal from alligators at either the Savannah National Wildlife Refuge (SNWR) or Par Pond in South Carolina, individual leeches were maintained in sterile 50-mL conical tubes containing 50 mL of 0.22-µm-filtered artificial freshwater medium [prepared in 20 L deionized water by dissolving sodium bicarbonate (0.89 mM, 1.5 g), calcium sulfate (0.063 mM, 0.17 g), and Jungle Aquarium Salt (0.0018% w/v; 0.36 g; Jungle Labs, Blacksburg, VA)] without access to a blood meal for up to 58 days. Leeches were maintained at 18 °C under static conditions without aeration or mixing but tubes remained open to the atmosphere to permit gas exchange. The 0.22-µm-filtered artificial freshwater medium was replaced periodically throughout the experiment to maintain water quality. Because specimens were held for several days prior to molecular analysis, “early captive” microbiomes refer to leeches analyzed 16–19 days after collection, but prior to prolonged captivity experiments (i.e. a captivity-conditioned baseline). For SNWR leeches, the “early captive” population is represented by those collected and preserved in ethanol on day 19 after capture, and the “late captive” population is represented by leeches preserved on days 26, 39, 51, or 58. For Par Pond, the “early captive” population was represented by leeches preserved in ethanol on day 16, and the “late captive” population included leeches preserved on days 20 and 46. Initial time-zero sampling was not possible due to delays associated with specimen shipment to the laboratory.

### Molecular Analysis

DNA was extracted from whole surface-sterilized leeches (vortexed vigorously after storage in 95% ethanol), except when separating anterior from posterior body regions. For most *Placobdella* species the crop begins at somite XIX, where they become triannulate and blood can be seen through the body wall. Thus, the delineation between anterior and posterior was visually assessed at this location. In some cases, testes and ovaries were specifically dissected and extracted. The Qiagen DNeasy Blood & Tissue Kit was used following the manufacturer’s protocol, and DNA concentrations were determined using Qubit fluorometric quantification. For the swab samples, an additional 200 µl of enzymatic lysis ATL buffer was added to the lysis mixture to submerge the swab tips.

**Fig. 2:**
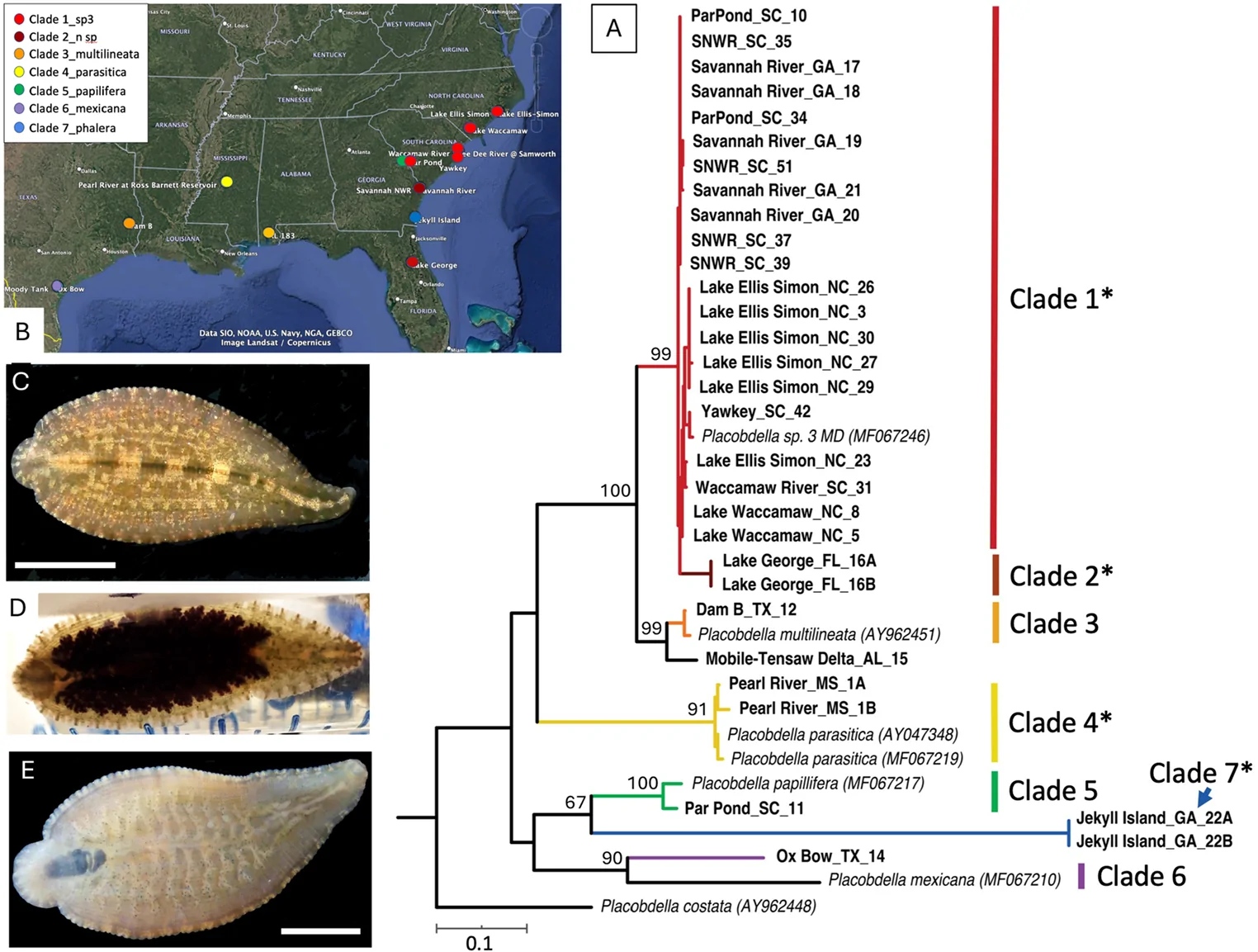
*Placobdella* specimens collected in this study. (**A**) Maximum likelihood phylogenetic reconstruction of the mitochondrial NADH dehydrogenase subunit 1 (ND1) gene using the GTR nucleotide substitution model. New sequences presented here are differentiated in bold. Clade 1 comprised 18 specimens collected from North Carolina, South Carolina, and Georgia that shared 98.3–100% ND1 sequence similarity with *Placobdella* sp. 3 previously reported from Maryland (red). Additional clades included two specimens of a likely undescribed *Placobdella* species from Florida most closely related to *Placobdella* sp. 3 (95.9–96.6% similarity; Clade 2, dark red), a specimen from Texas closely related to *P. multilineata* (99.3%; Clade 3, orange), two Mississippi specimens closely related to *P. parasitica* (98.9%; Clade 4, yellow), a single specimen from Par Pond, South Carolina most closely related to *P. papillifera* (96.6%; Clade 5, green), another specimen from Texas most closely related to *P. mexicana*, although highly divergent (83.7%; Clade 6, purple), and two specimens from Jekyll Island, Georgia representing a likely undescribed lineage distantly related to *P. phalera* (80.9%; Clade 7, blue). Branch support is indicated as bootstrap percentage, based on 1000 resamplings; only values > 60% are shown. Clades marked with an asterisk (*) have not been reported previously on alligators. **B** Geographic distribution of sampled *Placobdella* clades across the southeastern United States. Sampling locations are color coded according to the dominant *Placobdella* clade recovered at each site. **C** Representative whole specimens of *Placobdella* Clade 1 shown in dorsal view, (**D**) ventral view following a recent bloodmeal, and (**E**) ventral view after 58 days in captivity with substantial reduction in blood content. Scale bars, 1 cm

A fragment of the mitochondrial NADH dehydrogenase (ND1) gene was amplified from 42 leeches, using the LND300F/HND1932R primers, according to Light and Siddall [26]. Based on a recent description of 2 new *Placobdella* species by Phillips et al., [36], ND1 is more informative for species-level questions of glossiphoniid leeches than other genes, such as those for COI and 18 S rRNA. Thermal cycling conditions were as follows; 94 °C: 5 min, followed by 30 cycles: 94 °C, 45 °C, and 72 °C: 1 min each; and 5 min at 72 °C. Successful amplification products were sequenced directly using Sanger sequencing, via Quintara Biosciences Inc., CA, after which a phylogenetic analysis was conducted (Fig. 2). Closely related reference sequences were identified using BLAST searches against the NCBI database (www.ncbi.nlm.nih.gov). Sequences were aligned using Clustal Omega (in Geneious Prime 2025.1.2; Biomatters, Inc. San Diego, CA), and a maximum likelihood phylogeny was constructed in IQ-TREE [35] under the GTR nucleotide substitution model with maximum-likelihood-estimated base frequencies. The tree was visualized in iTOL v7.6 [25] and finalized in Illustrator. *Placobdella* ND1 sequences have been submitted to the GenBank database under accession number PZ503227 - PZ503280.

To characterize the bacterial community of leeches and swabs, the V4­V5 hypervariable region of the 16S rRNA gene was PCR amplified using bacterial primers [515F: GTGYCAGCMGCCGCGGTAA and 806R: GGACTACHVGGGTWTCTAAT [5] with Illumina adapters on the 5′ end (San Diego, CA), according to Dal Bó et al., [7]. Each PCR product was secondarily barcoded with Illumina NexteraXT index v2 Primers that included unique 8-bp barcodes, with NEB Q5 Hot Start High-Fidelity Mix at an annealing temperature of 66 °C for 11 cycles. Barcoded products were purified using Millipore-Sigma (St.Louis, MO) MultiScreen Plate MSNU03010 with a vacuum manifold and quantified using the QuantIT PicoGreen dsDNA Assay Kit (Thermo Fisher Scientific, Waltham, MA) on a BioRad CFX96 Touch Real-Time PCR Detection System. Barcoded samples were combined in approximately equimolar amounts and purified again with Promega’s Wizard SV Gel and PCR Clean-up System and quantified again using the QuBit system. A single sample, usually 1500–3000 ng DNA total, was submitted to Laragen, Inc. (Culver City, CA) for 2 × 300–bp paired-end analysis on the Illumina MiSeq platform with PhiX addition of 20%.

### Bioinformatic Analysis

Raw reads were processed according to Lee [24]. Briefly, CutAdapt v4.1 was used to remove primer sequences, which allowed one error for every 10 bp in the primer sequence [31]. FastQC v1.13 was used to quality control the raw sequence data and identify trim cutoffs for both the forward and reverse reads, ahead of pairing. Raw sequences were processed with DADA2 for initial quality trimming, error rate estimation, merging of read pairs, chimeric sequence removal, and community data matrix construction [4] and taxonomy was assigned to the processed amplicon sequence variants (ASVs at 100% identity) using the SILVA database v138.1, and confirmed using BLAST via GenBank. The raw Illumina 16S rRNA gene barcode sequences and metadata collected in this study are available from the NCBI Small Read Archive (BioProject #PRJNA1476394).

**Fig. 3:**
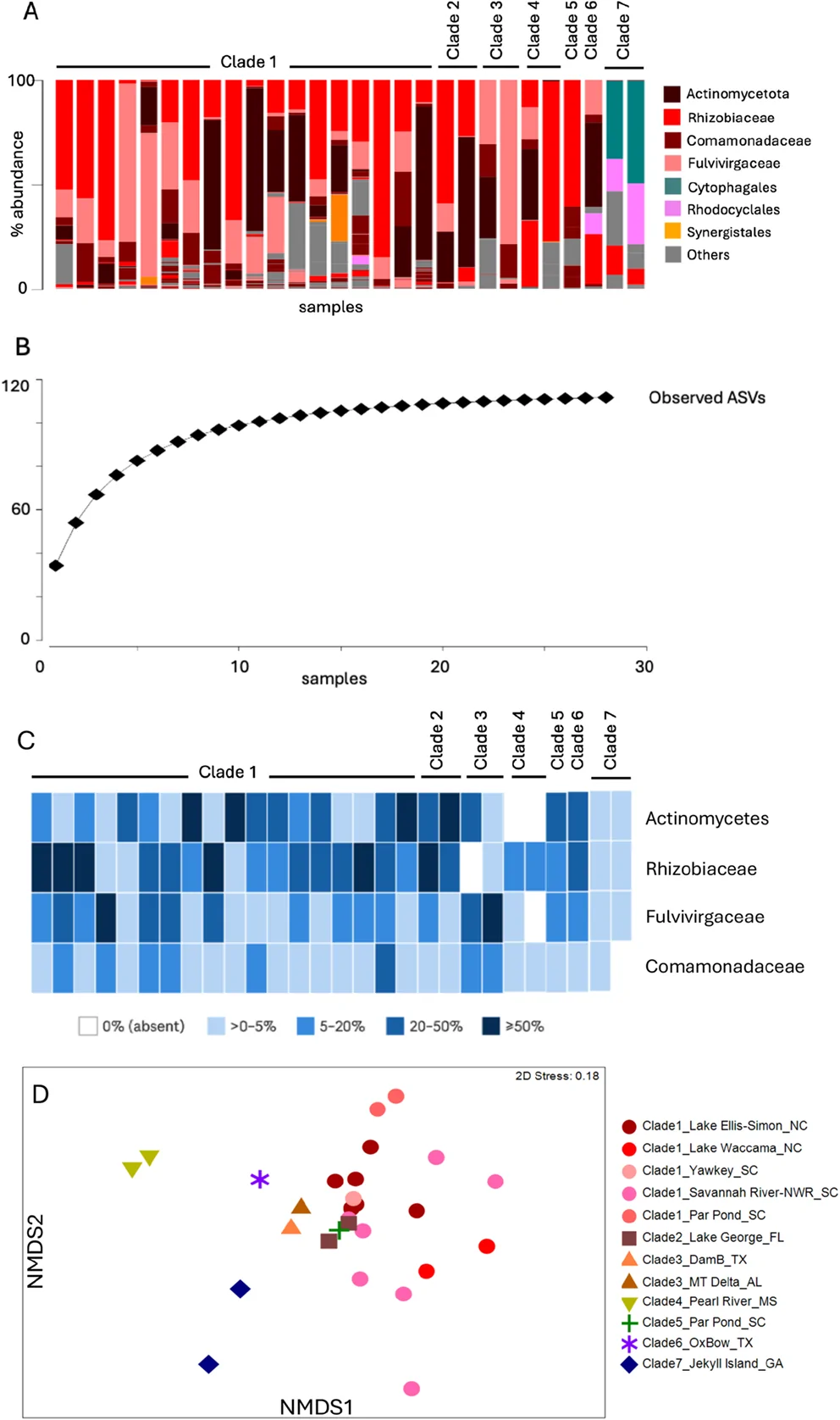
Broad microbiome composition and candidate core microbiome structure across *Placobdella* clades. **A** Relative abundances of dominant bacterial ASVs recovered from 28 *Placobdella* microbiomes representing seven host clades based on 16S rRNA gene sequencing. Each color represents a distinct bacterial ASV. Bacterial taxa are labeled at the lowest confidently assigned taxonomic rank based on 16S rRNA gene sequence classification; consequently, labels include a mixture of phylum-, order- and family-level assignments where finer taxonomic resolution was not supported. Bacterial taxa that constituted < 1% of sequences from individual specimens were grouped as “Other.” Samples are grouped according to host phylogenetic clade inferred from mitochondrial NADH dehydrogenase (ND1) sequences. **B** Species accumulation curve showing cumulative bacterial ASVs recovered from 28 *Placobdella* specimens analyzed. The asymptotic trends suggest that most of the bacterial diversity was captured within the current sampling effort. **C** Heatmap illustrating relative abundances of four candidate core taxa across all *Placobdella* specimens. Color intensity corresponds to relative abundance categories ranging from absence (0%) to dominant abundance (> 50%). Candidate core microbiome members were repeatedly recovered across multiple host clades and geographic locations. **D** Non-metric multidimensional scaling ordination of CLR-transformed community composition data of *Placobdella* microbiomes, based on 16S rRNA gene barcoding, with samples colored by leech clade, inferred from mitochondrial NADH dehydrogenase (ND1) phylogeny, and collection site

We define core microbiome using a combination of occurrence and abundance. According to Neu et al., [33], occurrence and abundance can each be used to quantify the core microbiome but combining them can provide a more conservative approach. For our study the candidate core microbiome is operationally defined as ASVs present in > 90% of wild specimens, which remained relatively stable or became enriched over the 58-day captivity period, persisting in captivity at > 5% relative abundance across the captive population. This prevalence threshold is similar to or greater than contemporary metrics for invertebrates [1, 41] and is consistent with the persistence-based concept proposed by Shade and Handelsman [42]. Additionally, maximizing sequencing depth is important for quantifying core microbiomes, especially when using strict occurrence cut-offs. Sequencing depth across samples ranged from 37,354 − 330,757 per sample for leeches and 38,883 − 142,212 for alligator skin samples. According to our datasets (ex. Figures 3B and 7C), this sequencing depth was sufficient to accurately capture the biological diversity present. Lastly, we defined the core microbiome using a 98% sequence similarity threshold, rather than exact sequence variants (100% identity) to reduce the influence of biologically insignificant sequence variation and obtain a more representative estimate of the shared core microbiome [33].

**Fig. 4:**
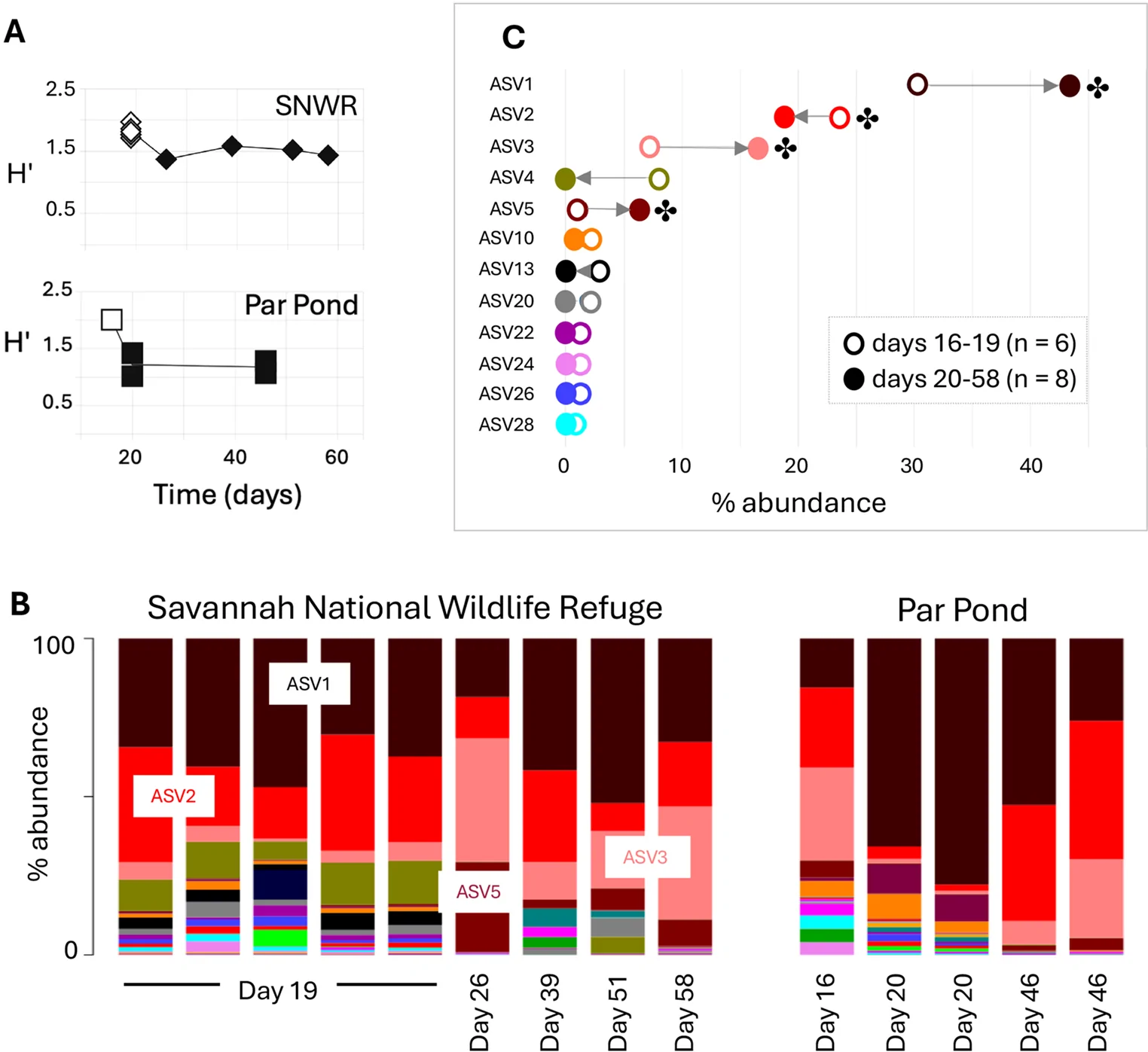
Microbiome comparisons between Placobdella specimens collected from Savannah National Wildlife Refuge (SNWR) and Par Pond specimens maintained in captivity without a blood meal for up to 58 days. **A** Shannon diversity (H′) of microbiomes from early captive specimens (open symbols) and late captive specimens (closed symbols) through time. **B** Relative abundances of bacterial ASVs recovered from individual leech microbiomes. Colors represent individual ASVs, as in Fig. 3. Four persistent ASVs identified as candidate core microbiome members are highlighted in white boxes. **C** Average relative abundances of the 12 ASVs contributing most strongly to microbiome differences between early and late captive SNWR specimens according to SIMPER analysis. Average dissimilarity between early and late captive SNWR microbiomes was 49.8%, driven by shifts in a subset of dominant ASVs. ASVs that remained relatively stable or became enriched during captivity are indicated by the clover-shaped symbol (✤) and were classified as candidate core microbiome members, whereas ASVs that declined to near-zero relative abundance during captivity were considered candidate transient microbiome members

### Statistical Analysis Metrics

To specifically estimate microbial diversity, the Shannon-Wiener Index (H′) was employed. Amplicon sequence variant (ASV) abundance data were centered log-ratio (CLR) transformed in R Studio (version 2024.12.1; [39]) prior to multivariate analysis. The CLR transformation was applied to account for the compositional nature of the ASV abundance data [16] and rarefaction was performed prior to CLR transformation to control for uneven sequencing depth across samples, consistent with recent recommendations [40]. To confirm this transformation choice did not alter our conclusions, we also compared raw-count Bray-Curtis and Euclidean approaches to CLR-transformed Euclidean (Aitchison) distance; all three yielded significant species effects (*P* = 0.001), with R2 ranging from 0.34 to 0.40. Nonmetric multidimensional scaling ordination (NMDS), similarity percentage analysis (SIMPER), and Permutational Multivariate Analysis of Variance (PERMANOVA) were performed in PRIMER-E (v. 7.0.25; [6]), using Euclidean distance matrices generated from the CLR-transformed ASV abundance data. For PERMANOVA+ analyses, Type III (partial) sums of squares and 999 permutations were used to evaluate differences in microbial community composition between groups. Pseudo-F indicates the strength of the signal relative to noise, given the degrees of freedom, while R² (calculated as SS(factor) / SS(Total), where SS = raw sum of squares) represents effect size and describes the proportion of variance explained by each factor. Permutational analysis of multivariate dispersions (PERMDISP) was performed to assess the homogeneity of within-group dispersions and to determine whether significant PERMANOVA results could be attributed to significant differences in within-group variance. One-tailed Mann Whitney U tests were performed using Social Science Statistics (www.socscistatistics.com).

## Results

### *Placobdella* Diversity and Phylogenetic Structure

Mitochondrial NADH dehydrogenase sequencing of 42 leeches identified seven *Placobdella* clades associated with American alligators (Fig. 2). Sampling included four lineages not previously reported from *Alligator mississippiensis*, including several likely undescribed taxa. Clade 1 comprised 18 specimens collected from North Carolina, South Carolina, and Georgia and shared 98.3–100% ND1 similarity with a specimen (*Placobdella* sp. 3) previously recovered from alligators in Maryland [9]. Additional clades included taxa related to *P. multilineata*, *P. parasitica*, *P. mexicana*, *P. papillifera*, and *P. phalera*, although several lineages were highly divergent from their closest described relatives (Fig. 2).

### Candidate Core Taxa Occur Across *Placobdella* Species

Bacterial barcoding of the 16S rRNA gene recovered 112 bacterial ASVs across 28 *Placobdella* specimens representing all seven clades (Fig. 3A-B). Four notably abundant candidate core bacterial taxa, however, were recovered from > 90% of the 28 specimens across the *Placobdella* lineages (Fig. 3C); including *Reichenowia*-associated Rhizobiaceae (Alphaproteobacteria; 22.9% average relative abundance), Actinomycetes classified primarily as *Jonesia* (20.8%), Fulvivirgaceae-associated Bacteroidota (15.1%), and Comamonadaceae-associated Betaproteobacteria classified as *Allobrachymonas* (7.4%). These taxa accounted for approximately 62% of all reads in the entire dataset. Identical or closely related (within 98%) ASVs of Actinomycetes, Rhizobiaceae, Fulvivirgaceae, and Comamonadaceae were repeatedly detected across multiple lineages despite broad phylogenetic and geographic separation among specimens. ASV1 accounted for > 99.4% of all Actinomycetes reads. ASV2 (*Reichenowia*) comprised 93.8% of all Rhizobiaceae. ASV3 comprised 98.3% of the total Fulvivirgaceae-associated reads. Lastly, ASV5 accounted for ~ 75% of all Comamonadaceae reads, with most of the remaining contributing less than 0.2% each.

To visualize patterns of whole-body microbiome variation across *Placobdella* clades and evaluate the influence of leech clade, geography, and hydrology, we examined community composition using non-metric multidimensional scaling (NMDS) ordination alongside PERMANOVA and PERMDISP analyses on CLR-transformed community composition data. PERMANOVA on the 5 *Placobdella* clades with *n* ≥ 2 suggested that leech clade significantly structured the microbiome (Pseudo-F = 2.74, *P* = 0.001; R2 = 0.34), however PERMDISP analysis indicated that significant within-clade heterogeneity contributed to the observed species effect (F = 8.07, *P* = 0.002), especially within Clade 1, which is consistent with their ecology; presence in many locations and habitats. Given this unequal dispersion, and the imbalance in sample sizes (range = 2–18), we are unable to fully resolve clade-level microbiome variation and this broad conclusion remains exploratory. Additionally, since each leech clade was represented at only a subset of locations (leaving empty cells in the design matrix), a 2-factor PERMANOVA design could not determine the effect of geolocation on microbiome composition. Generally, samples did not form discrete clusters by site, although some visual separation was apparent. Microbiomes associated with *P. phalera* from Jekyll Island, Georgia, and *P. parasitica* from Pearl River, Mississippi, occupied peripheral positions in space relative to the other five *Placobdella* clades (Fig. 3D; stress = 0.18). Microbiomes from Clade 1 specimens (*n* = 18) collected from both river and lake habitats were compared to evaluate whether broad hydrologic habitat influenced microbiome composition in *Placobdella*. No separation in microbiomes between these habitats of *Placobdella* was detected via PERMANOVA analysis (Pseudo-F = 1.10, *P* = 0.349, R2 = 0.13).

### Microbiome Shifts During Maintenance in Captivity Without Food

To assess microbiome stability during captive food-deprivation conditions, 14 additional *Placobdella* specimens from Clade 1 (> 99% identity based on ND1) were collected in 2025 from SNWR and Par Pond and maintained alive in laboratory aquaria for up to 58 days. By measuring the microbiome post-captivity, we decoupled the leeches from their normal external pool of bacteria; taxa that persist are unlikely to be there by chance. Microbiome diversity significantly decreased during captivity (H′ = 1.34 ± 0.20 SD; *n* = 8) compared to early captive specimens (H′ = 1.86 ± 0.11 SD; *n* = 6; Mann-Whitney U Test *P* = 0.001; Fig. 4A). PERMANOVA analysis revealed significantly altered microbial community composition during captivity (Pseudo-F = 4.94, *P* = 0.004, R2 = 0.29), and PERMDISP analysis supported this by failing to detect significant differences in dispersion between early and late timepoints (F = 0.68, *P* = 0.57). While captivity is itself a stressor that can trigger dysbiosis, especially if environmental parameters (i.e. temperature) are not maintained, captivity effects are not universal. Importantly, when dysbiosis occurs, it often produces stochastic, idiosyncratic changes in all directions [52]. In our study, we observed convergence on a new shared state by the captive leeches and there was no difference in dispersion between wild and captive populations. Thus, these retained taxa are not likely an introduction of new transients based on artificial conditions, but rather a retention of core microbes via possible selection exerted by the host leech, in the absence of a blood-meal.

**Fig. 5:**
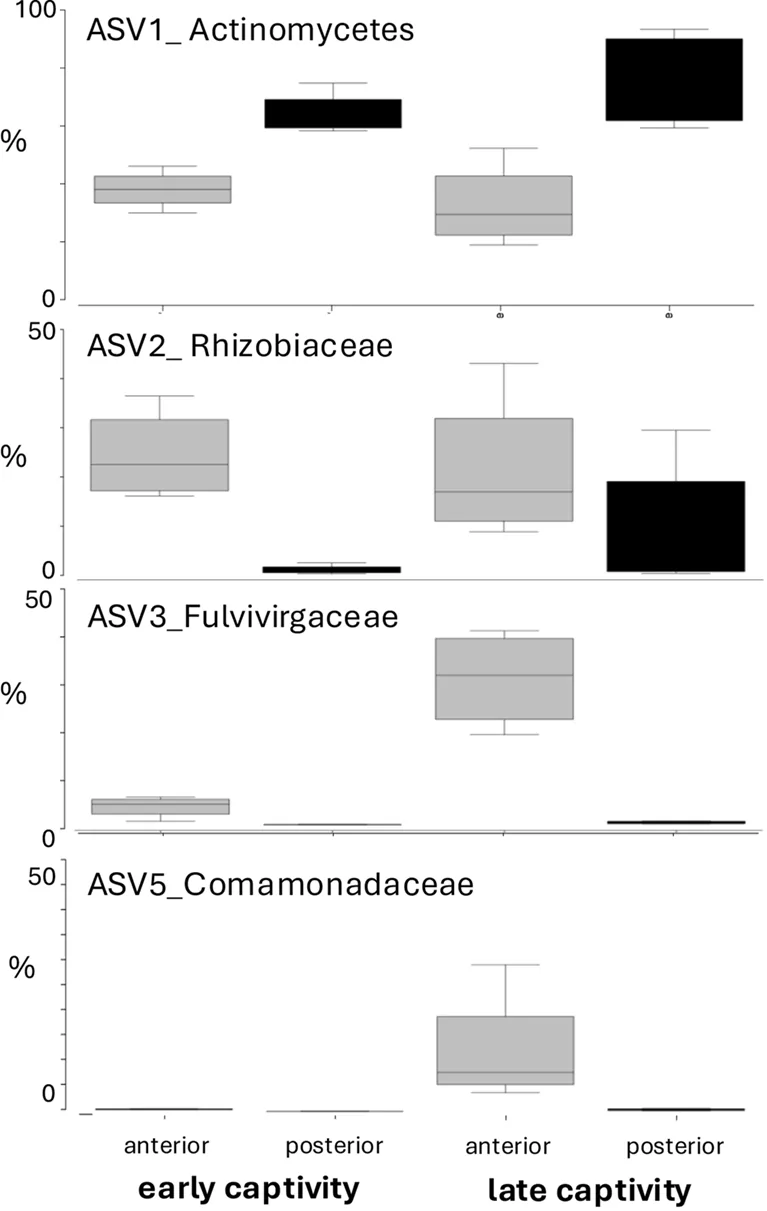
Relative abundances of the candidate core microbiome ASVs in *Placobdella* Clade 1 specimens dissected into anterior and posterior tissue regions from both early and late captive populations. ASV1 was distributed throughout both anterior and posterior tissues, whereas ASV2 (*Reichenowia*) was detected predominantly in anterior tissues of early captive specimens but extended into posterior tissues following prolonged captivity, and ASV3 and ASV5 were concentrated primarily in anterior tissues

Significant shifts in the *Placobdella* microbiome during captivity revealed persistence of the four candidate core microbiome members that either remained relatively stable or became enriched over the 58-day period (Fig. 4B-C). These included an Actinomycetes closely related to reptile-associated bacteria (ASV1; [46]), a *Reichenowia* ASV previously identified as a primary symbiont of *Placobdella* (ASV2; [43]), a member of the family Fulvivirgaceae related to those commonly recovered from lakes and rivers (ASV3; [3]), and a Comamonadaceae ASV also related to river taxa (ASV5; [17]). Collectively, these dominant ASVs comprised ~ 64% of the microbiome in early captivity leeches, a value similar to the wild caught leeches (~ 62%), highlighting consistency within the genus. Following the prolonged period of captive starvation, these 4 taxa comprised 87% of the microbiome (Fig. 5B). The relative proportion of some bacterial taxa declined during captivity, including ASV4 (related to Methylophilaceae), which comprised 9% of the microbiome initially and only 0.2% following captivity, as well as several others that collectively decreased in relative abundance across the population from ~ 5% to near 0% after prolonged captivity without food (Fig. 4C). These specific ASVs were considered to represent transient microbiome members consistent with taxa commonly associated with aquatic or sediment-associated environments. We note that the relative abundance changes described here reflect proportional shifts within the community and do not necessarily indicate absolute changes in ASV abundance, as an increase or decrease in one taxon’s relative abundance can result from changes in other, co-occurring taxa rather than a true change in that taxon alone.

**Fig. 6:**
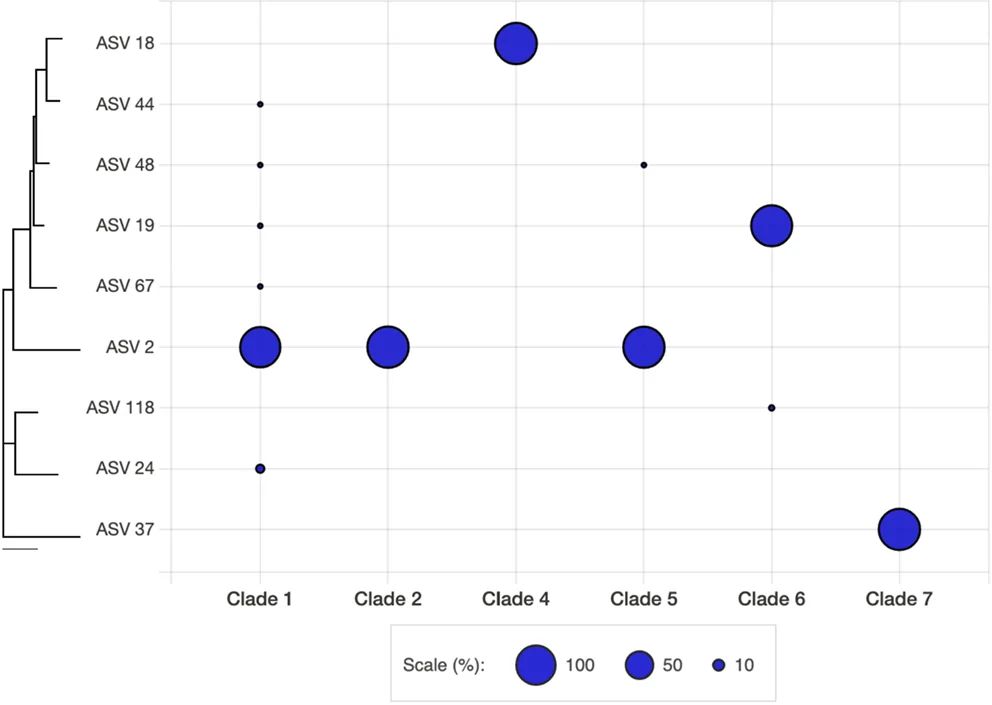
Relative abundances and phylogenetic relationships of *Reichenowia*-related ASVs recovered from six *Placobdella* clades based on 16S rRNA gene sequencing and organized according to host leech clade inferred from mitochondrial NADH dehydrogenase (ND1) sequences. Bubble size corresponds to relative abundance of individual ASVs within each *Placobdella* clade. ASV2 was recovered across multiple divergent *Placobdella* clades and represented the dominant *Reichenowia*-related ASV in Clades 1, 2, and 5. The alphaproteobacterial ASV associated with Clade 7, although classified as *Rhizobiaceae*, did not cluster within the primary *Reichenowia* clade. No *Reichenowia*-related ASVs were recovered from the single P. multilineata specimen analyzed. Left: neighbor-joining phylogenetic reconstruction of *Reichenowia*-related 16S rRNA ASVs generated using the Tamura–Nei substitution model

Several candidate core taxa also exhibited consistent localization patterns within the leech body (Fig. 5). For example, ASVs 3 and 5 were concentrated primarily in anterior tissues containing the esophagus, salivary glands, and mycetomes, whereas ASV1 (Actinomycetes) was detected throughout the digestive tract, including the crop and intestine. *Reichenowia* (ASV2) was detected primarily in anterior tissues of early captive leeches but extended into posterior regions following prolonged captivity (Figs. 5 and 6). While the microbiome data presented here represents the whole-body-associated microbiome of *Placobdella* (i.e. the gastrointestinal tract was not dissected), these findings suggest that microbial community structure within the leech body may not be uniform, even at this coarse spatial resolution.

### *Reichenowia* Diversity and Symbiont Sharing Across *Placobdella*

A distinct dominant *Reichenowia* symbiont was recovered from four of the six *Placobdella* clades (Fig. 6, broadly consistent with previous hypotheses of long-term host association and vertical transmission. However, the dominant *Reichenowia* ASV2 was identical in both Clades 1 and 2, despite the host lineages differing by 3–4% in the ND1 gene (Fig. 2). Additionally, the Clade 6 (*P. papillifera*) specimen living in Par Pond sympatric with Clade 1 hosted an identical dominant *Reichenowia* ASV despite belonging to a more distantly related host lineage (17% ND1 divergence; Fig. 2). This raises the question of environmental acquisition, as do the five additional low-abundance *Reichenowia* ASVs recovered from Clade 1 specimens (Fig. 6). Similarly, single individuals from Clades 6 and 7 also housed additional low abundance *Reichenowia* ASVs. No *Reichenowia*-related ASVs were recovered from the single *P. multilineata* specimen analyzed. Unlike Manglicmot et al., [30], attempts at DNA amplification of *Reichenowia* from testisacs and ovaries, was negative for Clade 1.

**Fig. 7:**
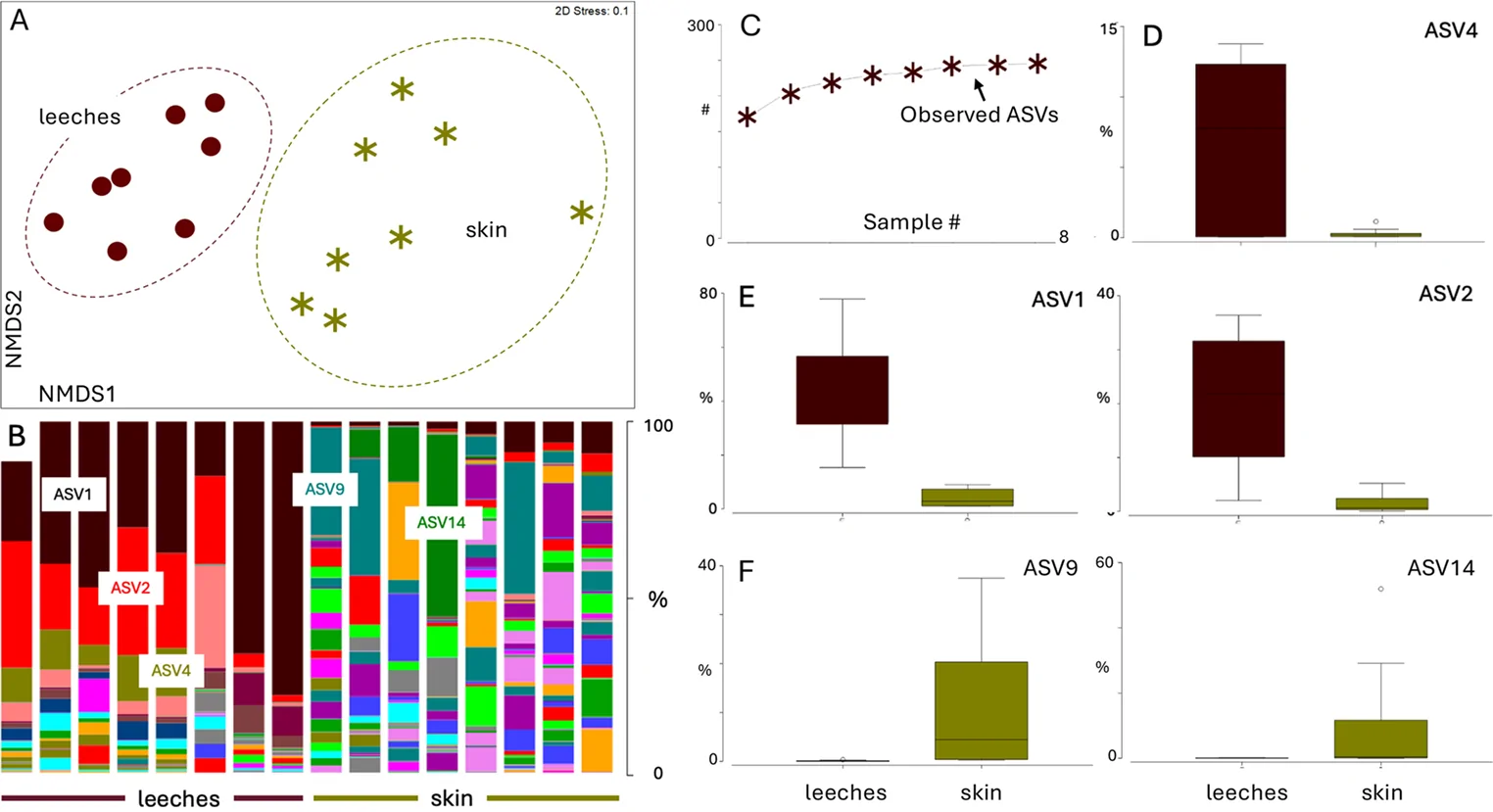
Comparisons between *Placobdella* and alligator skin microbiomes. **A** Non-metric multidimensional scaling (NMDS) ordination illustrating microbial community similarity between *Placobdella* Clade 1 microbiomes and microbial communities sampled from alligator skin [stress = 0.1]. NMDS separation was supported by PERMANOVA analysis. **B** Relative abundances of bacterial ASVs recovered from leech and alligator skin microbiomes (*n* = 8 per group). Colors represent individual ASVs. **C** Species accumulation curve showing cumulative bacterial ASVs recovered from 8 alligator skin samples. **D** Relative abundances of representative bacterial ASVs differing between leech and skin microbiomes. ASV4 was detected primarily within leech microbiomes. **E** Candidate core microbiome members ASV1 and ASV2 were detected in both leech and skin samples, although substantially enriched within leeches. **F** ASV9 and ASV14 were associated primarily with alligator skin microbiomes

### Comparison Between Leech and Alligator Skin Microbiomes

To assess a potential environmental reservoir for leech-associated bacteria, microbiomes from Clade 1 *Placobdella* specimens were compared with microbial communities sampled from alligator skin at Savannah National Wildlife Refuge and Par Pond. NMDS ordination of CLR-transformed community composition data revealed separation between leech-associated and skin-associated microbiomes (Fig. 7A-B; *n* = 8 per group). This separation was supported by PERMANOVA analysis (Pseudo-F = 8.64, *P* = 0.001, R2 = 0.38), indicating that *Placobdella* harbored significantly distinct microbial communities relative to alligator skin. Microbiome diversity was significantly greater on alligator skin (H′ = 2.64 ± 0.38 SD) than within leeches (H′ = 1.71 ± 0.36 SD; Mann-Whitney U Test *P* = 0.0008). SIMPER analysis indicated that the average pairwise Bray-Curtis similarity among leech microbiomes (~ 62%) was greater than among alligator skin microbiomes (~ 49%). Overall dissimilarity between leech and skin microbiomes was approximately 75% based on SIMPER analysis. Two candidate core taxa, Actinomycetes (ASV1) and *Reichenowia* (ASV2), were recovered at low abundance from alligator skin (Fig. 7C). Several additional ASVs associated with microorganisms commonly reported from environmental sources were detected exclusively on alligator skin (Fig. 7C).

## Discussion

This study sought to characterize the microbiome of *Placobdella* leeches collected from American alligators and to evaluate potential drivers of microbiome composition across host lineages, geographic regions, and habitat types. Within seven *Placobdella* clades, many of which have not been reported on alligators, we identified a diverse bacterial assemblage that consistently included the well-described alphaproteobacterial symbiont *Reichenowia*, along with a broader suite of taxa spanning Actinomycetes, Bacteroidota, and Pseudomonadota (the former Betaproteobacteria). These results extend prior work focused primarily on *Reichenowia* and indicate that *Placobdella* harbor more complex microbial communities than previously appreciated.

*Placobdella* microbiome composition was significantly structured by leech clade although it remains unclear whether this pattern reflects a genuine host effect or the sample size imbalance across clades (*n* = 2–18). Expanded sampling of underrepresented clades would help resolve this. Geolocation, by contrast, showed no clear signal, although certain clades were only sampled from certain locations. Some differences associated with sampling location were apparent, with distinct microbiomes recovered from *P. phalera* from Georgia and *P. parasitica* from Mississippi, however without paired environmental sampling, we cannot determine whether these taxa reflect environmental exposure or a stable component of their microbiomes. Variation in leech attachment sites on alligators (e.g., mouth, limbs, dorsal surfaces) may also introduce heterogeneity in microbial exposure. Together, these findings suggest that microbiome assembly in *Placobdella* may be influenced by fine-scale ecological and host-associated factors not fully resolved in this study.

Consistent with previous work, *Reichenowia* was detected in the majority of *Placobdella* species-clades examined, and each host lineage generally harbored a dominant *Reichenowia* variant. This pattern supports expectations for vertically transmitted symbionts, which are typically maintained with high fidelity across host lineages [30, 43]. However, observations of individual leeches harboring multiple low-abundance *Reichenowia* variants (also observed by [30]) and identical 16S rRNA gene sequences detected in different *Placobdella* clades collected from the same location suggest the possibility that environmental exposure or occasional horizontal acquisition contributes to symbiont diversity. This pattern has been observed in other symbiotic systems where predominantly vertical transmission is supplemented by rare environmental uptake [10, 45]. *Reichenowia* forms a monophyletic group within the Rhizobiaceae, a lineage also known to establish environmentally-acquired symbioses with plants through horizontal transmission into root cells [15], further supporting the possibility that occasional environmental acquisition contributes to symbiont diversity in *Placobdella*.

Comparisons between leech microbiomes and microbial communities sampled from alligator skin revealed clear differences in composition and diversity, with relatively limited overlap. Similar patterns have been observed in other host-associated systems, where internal microbial communities are shaped by host selection and maintenance rather than direct environmental exposure [1, 41]. Yet, the presence of a small subset of shared taxa, including *Reichenowia*, indicates that the environment, in this case alligator skin bacteria, could contribute to *Placobdella* microbiome assembly. Other than alligator skin, this study did not include paired environmental samples, limiting our ability to distinguish potential environmental carryover. Thus, more comprehensive sampling of environmental reservoirs (e.g. water column, sediment, and even alligator blood) will be necessary to clarify the sources and transmission of both core and transient taxa.

Temporal changes in the microbiome during laboratory maintenance of specimens within *Placobdella* Clade 1 provide insight into community stability. Microbial diversity decreased significantly during captive fasting, and community composition shifted within days, with some taxa declining in relative abundance while others persisted and/or increased in relative abundance. Following prior work in host-associated microbiomes, we use a combination of occurrence, and abundance under perturbation, to identify candidate core taxa. Microbes that remain relatively stable or enrich during captivity, or upon dietary shifts, have been interpreted as more tightly associated with the host, whereas those that are rapidly lost are often considered environmentally derived or transient [33, 42]. Within this framework, the bacterial taxa that persisted across all time points (ex. *Reichenowia*, Actinomycetes, Fulvivirgaceae and Comamonadaceae) likely represent the stable core microbiome of *Placobdella*. The Actinomycetes identified in the *Placobdella* microbiome is closely related to bacteria previously associated with reptilian disease [46], highlighting the need for future investigation into the potential ecological and epidemiological roles of leech-associated bacteria in American alligator populations. Because our analyses are based on relative 16S rRNA gene sequence abundances, the persistence of candidate core taxa should be interpreted as persistence within the microbial community composition, rather than evidence of stable or increasing absolute abundance. Future studies incorporating quantitative estimates of bacterial load could determine whether these taxa are maintained numerically or become relatively enriched as other community members decline during prolonged starvation.

Blood-feeding organisms face physiological challenges including limited availability of essential nutrients and the need to process heme-rich diets [11, 29]. For example, some leeches lack key digestive enzymes and may therefore rely on bacterial symbionts to aid digestion [20]. *Reichenowia*, although known to aid in B vitamin production [23], cannot participate in the digestion of red blood cells or detoxification of heme, which would primarily occur in the crop and intestine, due to their localization to the anterior mycetomes [43]. For other blood-feeding parasites, investigators have suggested that additional microbes could be a potential novel heme detoxification strategy [47]. In the present study, several candidate core taxa are phylogenetically related to bacteria known to participate in iron cycling or organic matter degradation [3, 17, 27, 38]. While direct evidence for roles in heme detoxification, red blood cell breakdown, or nutrient provisioning is still required, these findings raise the possibility that *Placobdella* leeches maintain a functionally diverse microbiome that might contribute to host fitness.

## Conclusion

Collectively, our results indicate that *Placobdella* leeches harbor diverse and dynamic microbial communities that extend beyond the well-characterized *Reichenowia* symbiont. The *Placobdella* microbiome included both stable candidate core taxa that persisted during prolonged food-deprived captivity, alongside variable taxa likely acquired from environmental exposure to alligator skin and other reservoirs. While our sampling strategy did not cover the entire geographic distribution of *Placobdella*, the repeated recovery of four bacterial taxa across multiple clades, disparate geographic regions, and distinct ecological conditions supports the existence of a conserved candidate core microbiome within the genus. Further, while *Reichenowia* associations are generally conserved within *Placobdella*, our results suggest that symbiont sharing and occasional environmental acquisition likely occur. Together, these findings expand our understanding of microbiome assembly and symbiont persistence in blood-feeding leeches and establish *Placobdella* as a tractable system for investigating the ecological and evolutionary dynamics of host-associated microbiomes.

## Data Availability

The metadata and raw sequencing files generated and analyzed during the current study are available on the NCBI SRA repository under BioProject PRJNA1476394. Placobdella ND1 sequences have been submitted to the GenBank database under accession number PZ503227-PZ503280. All other data generated or analyzed during this study are included in this published article.
